# RBM47 is a novel immunotherapeutic target and prognostic biomarker in gliomas

**DOI:** 10.1038/s41598-024-84719-z

**Published:** 2025-01-05

**Authors:** Wei Wei, Yongfu Cao, Xin Lu, Long Wang, Jianbin Li, Guojun Deng, Donghai Li, Limin Xiao

**Affiliations:** 1 Department of Neurosurgery, The First Affiliated Hospital, Jiangxi Medical College, Nanchang University, No. 17 Yongwai Street, Nanchang, 330006 Jiangxi China; 2 Department of Neurosurgery, Key Laboratory of Biological Targeting Diagnosis, Therapy and Rehabilitation of Guangdong High Education Institutes, The Fifth Affiliated Hospital, Guangzhou Medical University, Guangzhou, Guangdong China; 3 Department of Neurosurgery, Jiangxi Cancer Hospital & Institute, Jiangxi Clinical Research Center for Cancer, The Second Affiliated Hospital of Nanchang Medical College, Nanchang, Jiangxi China

**Keywords:** Glioma, RBM47, Immunotherapy, Immune microenvironment, Macrophage, Computational biology and bioinformatics, Immunology, Biomarkers, Oncology

## Abstract

The role of RBM47, an RNA-binding protein, in shaping the immune landscape of gliomas and tumor immune responses is yet to be fully studied. Therefore, a comprehensive investigation into the immunomodulatory roles of RBM47 in gliomas was conducted, leveraging gene expression data from multi-omic datasets. The prognosis of patients with gliomas considering RBM47 was elucidated using bioinformatics methods and clinical data, with results validated using immunohistochemistry and immunofluorescence analyses. The expression of RBM47 in gliomas was higher than that in normal tissues and was positively correlated with the World Health Organization tumor grade. Increased RBM47 expression is associated with poor prognosis in patients with glioma, serving as an independent predictor of overall survival. The nomogram combining RBM47 expression levels with clinical prognostic factors demonstrated strong predictive accuracy, achieving a C-index of up to 0.863 in both TCGA training and CGGA validation groups. Gene Ontology, Kyoto Encyclopedia of Genes and Genomes, and Gene Set Variation Analysis indicated that RBM47 is closely related to immunity and inflammation. Single-cell sequencing and immunofluorescence assays confirmed the enrichment of RBM47 in CD163 + macrophages. Therefore, RBM47 plays a vital role in the immune microenvironment of gliomas and may be a potential immunotherapy target.

## Introduction

Gliomas, which are the most prevalent malignant brain tumors in the central nervous system, represent a severe threat to human health^1^. The main treatment methods include surgical resection, postoperative radiotherapy, and chemotherapy^2,3^. Given the short overall survival and poor prognosis of patients with glioma under current treatment strategies, the need to explore novel molecular targets is urgent.

In recent years, research has uncovered potential immunotherapy targets for several types of tumors^4,5^, offering new prospects for tumor treatment. Immunotherapy has yielded optimistic results in the treatment of various solid tumors such as melanoma, lung cancer, and Hodgkin’s lymphoma^6–8^. While previous studies have identified potential immunotherapeutic targets in glioblastoma^9–11^, few immune checkpoint inhibitors have proven effective in enhancing the prognosis of patients diagnosed with glioblastoma^3,12,13^. Therefore, extensive research on the tumor-immunosuppressive microenvironment of gliomas, novel immunotherapy targets, and the enhancement of patient prognosis is urgently required.

RBM47 is a multifunctional protein belonging to the diverse family of RNA-binding proteins (RBPS). RBPS can promote cancer progression by influencing post-transcriptional stages of RNA processing, including translation, splicing, stability, and localization^14,15^. Research has shown that RBM47 plays an important role in the progression of various cancers, including colorectal cancer, thyroid cancer, hepatocellular carcinoma, and nasopharyngeal cancer. RBM47 can inhibit the migration, invasion, and proliferation of renal cancer cells^16^; it acts as a regulatory factor in the occurrence of intestinal tumors^17^, and it regulates thyroid cancer cell proliferation through the RBM47/SNHG5/FOXO3 axis^18^. This demonstrates that its role may vary across different tumor types. Recently, RBM47 has been discovered to play an essential role in immune cells. For example, RBM47 overexpression enhances IL-10 production in B cells^19^. Moreover, RBM47 negatively regulates zebrafish-specific interferons^20^. According to previous studies, dioscin acts on RBM47 to modulate the immune microenvironment^21^, while RBM47 has been shown to promote the invasiveness of glioblastoma^22^. However, the role of RBM47 in glioma and its impact on tumor immune responses has yet to be thoroughly investigated.

Here, we systematically investigated the expression, immune effects, and the influence of RBM47 on the clinical prognosis of patients with gliomas using clinical samples and RNA sequencing (RNA-seq) data from GSE4290, the China Glioma Genome Atlas (CGGA), The Cancer Genome Atlas (TCGA), CGGA single-cell sequencing data, and the GSE214966 and GSE131928 datasets. Furthermore, we analyzed the relationship between the expression of RBM47 and crucial immune-related factors. We employed bioinformatics analyses to investigate the role of RBM47 in the immune process of gliomas. Immunostaining confirmed the correlation between RBM47 and M2 subtype macrophages, suggesting a close relationship between RBM47 and the immune microenvironment of tumors. Our findings suggest that RBM47 holds promise as both an independent prognostic factor and a potential immunotherapy target.

## Methods

### Data collection

The RNA-seq data and clinical information used in this study were derived from several datasets, including 702 samples from TCGA (http://cancergenome.nih.gov/,version 2022), 325 samples from CGGA (http://www.cgga.org.cn), and 180 samples from the GSE4290 dataset sourced from the Gene Expression Omnibus (GEO) repository (http://www.ncbi.nlm.gov/geo). Single-cell sequencing data were sourced from CGGA (http://www.cgga.org.cn), and the GSE214966 and GSE131928 datasets (GSM3828672) were sourced from the GEO repository. Detailed clinical information of the patients is shown in Table 1.

**Table 1:** Sample information.

Characteristics (CGGA2022)	Patients (n = 325)	Characteristics (TCGA)	Patients = 702
Gender		Gender	
Male	203	Male	354
Female	122	Female	255
WHO grade		NA	93
Grade II	103	WHO grade	
Grade III	79	Grade II	216
Grade IV	139	Grade III	241
NA	4	Grade IV	152
IDH status		NA	93
Mutation	175	IDH status	
Wildtype	149	Mutation	428
NA	1	Wildtype	234
1p/19q status		NA	40
Codeletion	67	1p/19q status	
Non-codeletion	250	Codeletion	169
NA	8	Non-codeletion	495
MGMT status		NA	38
Methylation	157	MGMT status	
Unmethylation	149	Methylation	492
NA	19	Unmethylation	168
		NA	42

### GO and KEGG analyses

The CGGA and TCGA databases were used to screen for the top 500 genes most closely related to RBM47, based on Pearson correlation analysis (*R* > 0.5, *P* < 0.01), arranged in ascending order of *P*-value. The gene list was submitted to the Database for Annotation, Visualization, and Integrated Discovery (https://davidbioinformatics.nih.gov/) for functional annotation and enrichment analysis. Subsequently, the resulting GO terms were subjected to Spearman’s correlation analysis, and the outcomes were visually represented using a heatmap.

### GSVA enrichment analysis

GSVA was applied as previously described using the GSVA package (1.52.3)^23,24^. The GO gene list was downloaded from the AmiGO 2 Web portal (http://amigo.geneontology.org/amigo/landing, c5.go.v2023.2.Hs.symbols.gmt), and charts were generated using Excel (Microsoft Corporation, Redmond, WA, USA).

### Single-cell data analysis

Single-cell data analysis followed established methods described in previous studies^25^. Briefly, downstream analysis was conducted using the Seurat package (version 5.1.0) to remove genes with nFeature-RNA > 200 or percent.mt < 20. Normalization was performed using the NormalizeData function, and the top 2,000 highly variable genes were selected for analysis. The ScaleData function was used for scaling, the RunPCA function was used for dimensionality reduction, and the FindNeighbors and FindClusters functions were used for cell clustering.

### Kaplan–Meier analysis

The Kaplan–Meier survival curve and COX regression analysis were used to examine the prognostic value of RBM47 across all glioma grades using the packages ggplot2 (3.5.1), ggpubr (0.6.0), survminer (0.4.9) and survival (3.7–0). Logarithmic rank *P*-values and hazard ratios with 95% confidence intervals were calculated.

### IHC and immunofluorescence analysis

IHC for RBM47 was conducted on paraffin-embedded sections of glioma and normal brain tissue, according to standard procedures. Briefly, antigens were extracted from glass slides in citric acid buffer (pH 6.0), after which the sections were incubated with a primary antibody against RBM47 (Bs-19774R, 1:100; Bioss Antibodies, Woburn, MA, USA) overnight at 4 °C. Thereafter, the samples were incubated with a secondary antibody (goat anti-mouse IgG, GB23301, 1:200; Servicebio, Wuhan, China). Subsequently, staining was performed using diaminobenzidine tetrachloride and hematoxylin. Multiple immunofluorescence staining was performed on the paraffin-embedded glioma sections using primary antibodies against RBM47 (Bs-19774R, 1:200; Bioss Antibodies), CD163 (TA506391S, 1:100; Origene Technologies, Inc., Rockville, MD, USA), goat anti-mouse IgG (BA1031, 1:100; Boster Bio, Pleasanton, CA, USA), and goat anti-rabbit IgG (BA1102, 1:100; Boster Bio), following standard procedures.

### Statistical analysis

Charts and statistical analyses were performed using R software (version 4.4.0; R Foundation for Statistical Computing, Vienna, Austria). Unpaired samples were subjected to the Wilcoxon rank-sum test. The Wilcoxon signed rank test and logistic regression were used to determine the association between clinical features and RBM47 expression. Statistical significance was considered at *P* < 0.05.

## Results

### Expression level of RBM47 in patients with glioma

**Fig. 1:**
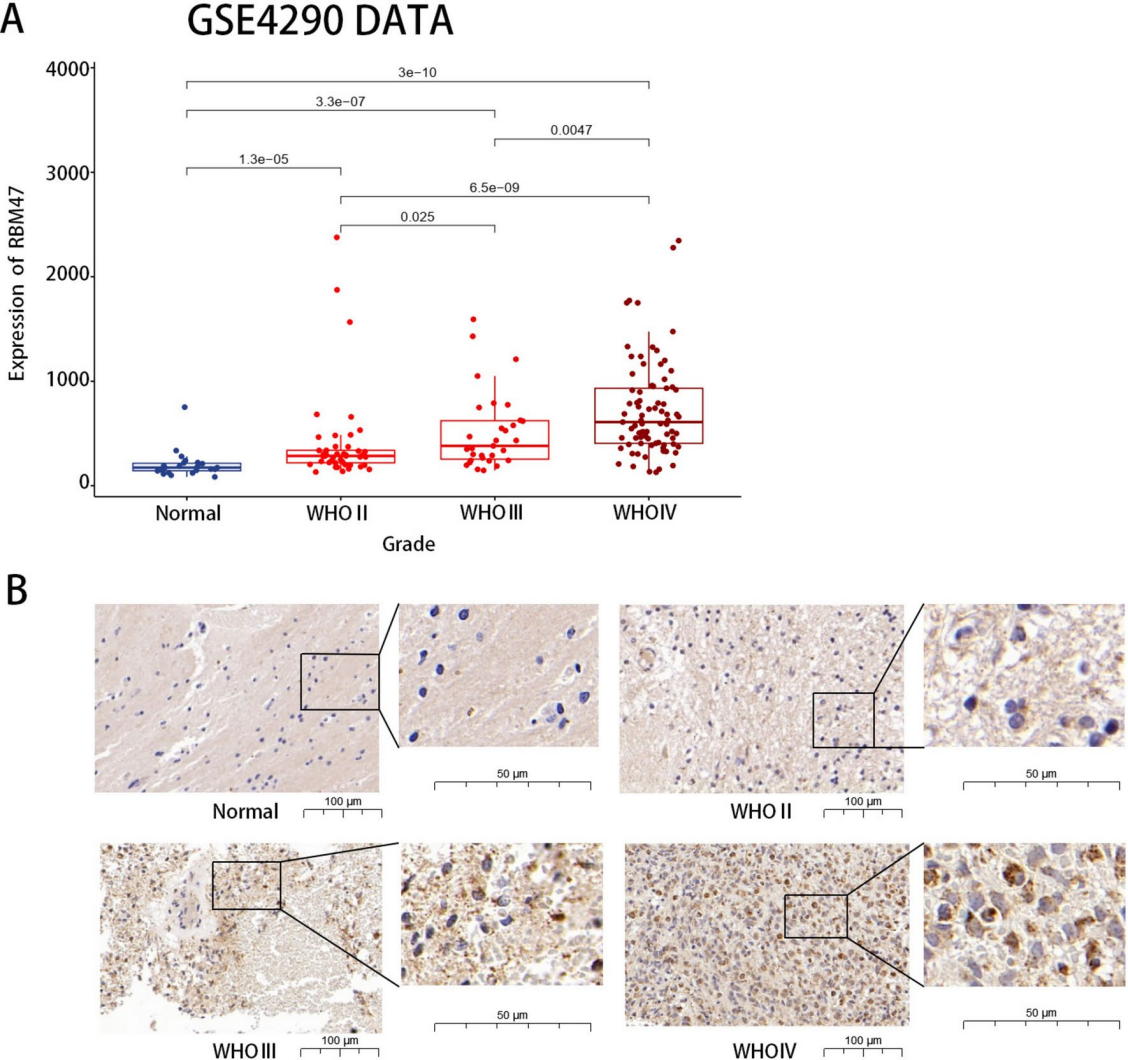
Expression of RBM47. (**A**) RNA-seq of the GSE4290 dataset showed that RBM47 expression was higher in tumors than in normal tissues and positively correlated with glioma grade. (**B**) Immunohistochemical validation confirmed that the protein expression of RBM47 is consistent with RNA-seq data. Sample sizes in the dataset: Normal *n* = 23, WHO II *n* = 45, WHO III *n* = 31, WHO IV *n* = 81. The differences between groups were analyzed using the Wilcoxon rank-sum test. *P* < 0.05 was considered significantly different.

We observed significantly higher RBM47 expression levels in glioma tissue compared with those in normal tissues, as evidenced by GSE4290 data. Furthermore, RBM47 expression positively correlated with increasing World Health Organization (WHO) grades (Fig. 1A). We validated the differences in RBM47 protein expression levels across gliomas of different grades and normal tissues through immunohistochemistry (IHC) (Fig. 1B). The immunohistochemical results were consistent with GSE4290 data.

### Relationship between RBM47 expression and clinical characteristics of the patients

**Fig. 2:**
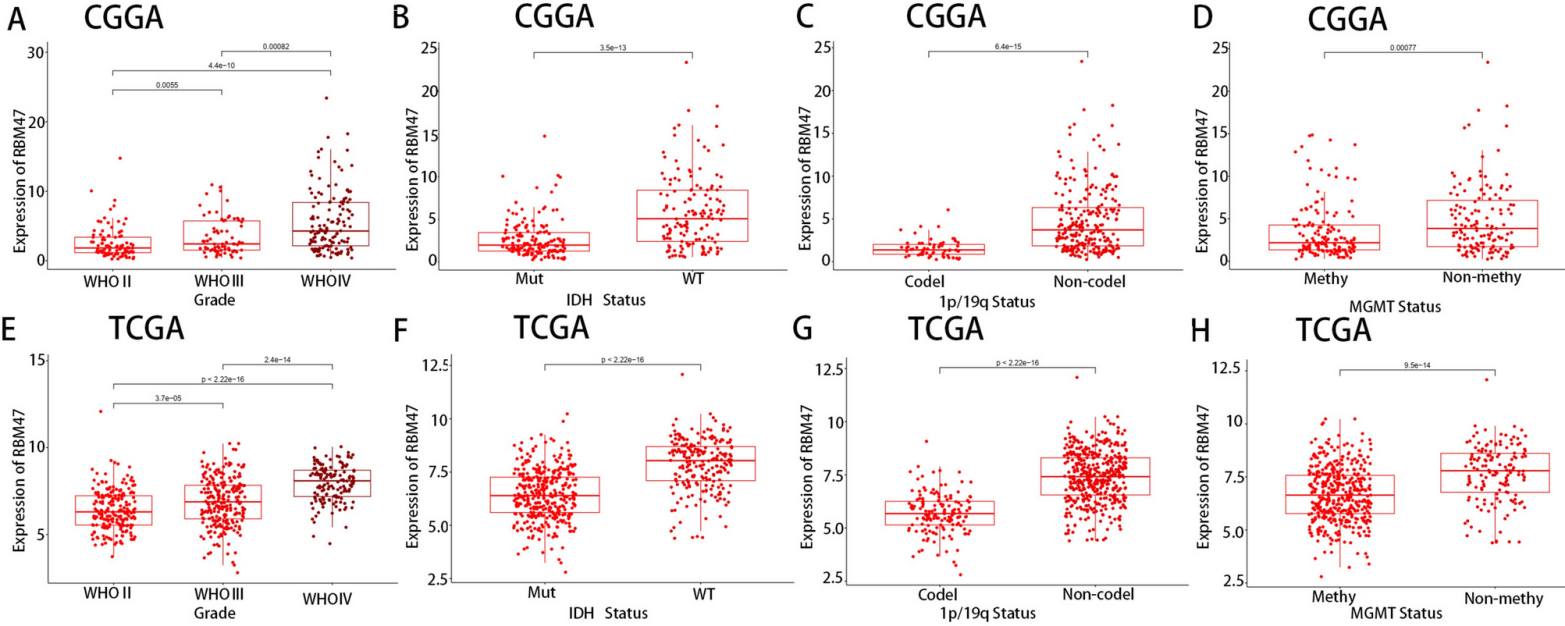
Relationship between RBM47 and clinical pathological features. (**A**–**D**) Relationship between RBM47 expression and WHO grade, IDH status, 1p/19q co-deletion, and MGMT status in the CGGA dataset. (**E**–**H**) TCGA dataset yielded similar results. Sample sizes in the CGGA dataset: WHO II *n* = 97; WHO III *n* = 73; WHO IV *n* = 134; IDH mutant type *n* = 163; IDH wild type *n* = 141; 1p/19q co-deletion *n* = 62; 1p/19q non-co-deletion *n* = 242; DNA methylated *n* = 147; DNA un-methylated *n* = 139. Sample sizes in TCGA dataset: WHO II *n* = 211; WHO III *n* = 238; WHO IV *n* = 143; IDH mutant type *n* = 372; IDH wild type *n* = 220; 1p/19q co-deletion *n* = 149; 1p/19q non-co-deletion *n* = 443; DNA methylated *n* = 419; DNA un-methylated *n* = 143. The differences between groups were analyzed using the Wilcoxon rank-sum test. *P* < 0.05 was considered significantly different.

We investigated the relationship between RBM47 expression and several clinical features in patients with glioma using RNA-seq data from TCGA and CGGA. In the CGGA database, RBM47 expression demonstrated an upward trend, correlating with increases in glioma grade (Fig. 2A), consistent with the results of GSE4290 data. In addition, RBM47 expression was notably higher in isocitrate dehydrogenase (IDH)-wild-type glioma samples, those without 1p/19q co-deletion, and those devoid of methylguanine-DNA methyltransferase promoter methylation (Fig. 2B–D). These results were validated using the TCGA database (Fig. 2E–H), and they suggest that RBM47 is highly expressed in highly malignant gliomas.

### RBM47 is correlated with immune functions in glioma

**Fig. 3:**
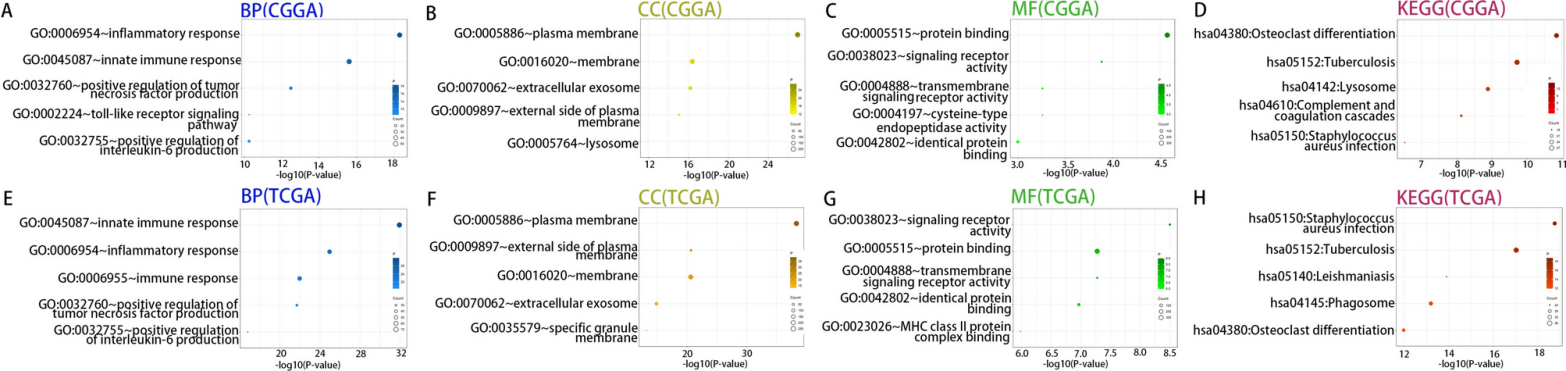
RBM47 is related to immune regulation. (**A–D**) Biological processes (BP), cellular components (CC), molecular functions (MF), and Kyoto Encyclopedia of Genes and Genomes (KEGG) analysis for RBM47 in the CGGA dataset. (**E–H**) TCGA dataset validation results.

Based on the identified genes, Gene Ontology (GO) and Kyoto Encyclopedia of Genes and Genomes (KEGG) analyses were performed to explore the biological functions of RBM47. In the CGGA database, the biological processes most relevant to RBM47 included the innate immune response, inflammatory response, positive regulation of tumor necrosis factor production, and positive regulation of interleukin-6 production (Fig. 3A). The most relevant cellular components were the plasma membrane and the membrane (Fig. 3B). The molecular functions included protein binding, signaling receptor activity, and transmembrane signaling receptor activity (Fig. 3C). The signaling pathways most significantly associated with RBM47 were those implicated in the host immune responses against tuberculosis and *Staphylococcus aureus* infections. These signaling pathways are intimately linked to immunity and inflammation, hinting at a possible function of RBM47 in regulating the body’s protective responses against bacterial infections (Fig. 3D). Comparable findings were replicated in the TCGA database (Fig. 3E–H), reinforcing the notion that RBM47 plays a pivotal role in modulating immunity, inflammation, immune-mediated disorders, as well as the intricate glioma microenvironment.

### RBM47 is related to the immune response

**Fig. 4:**
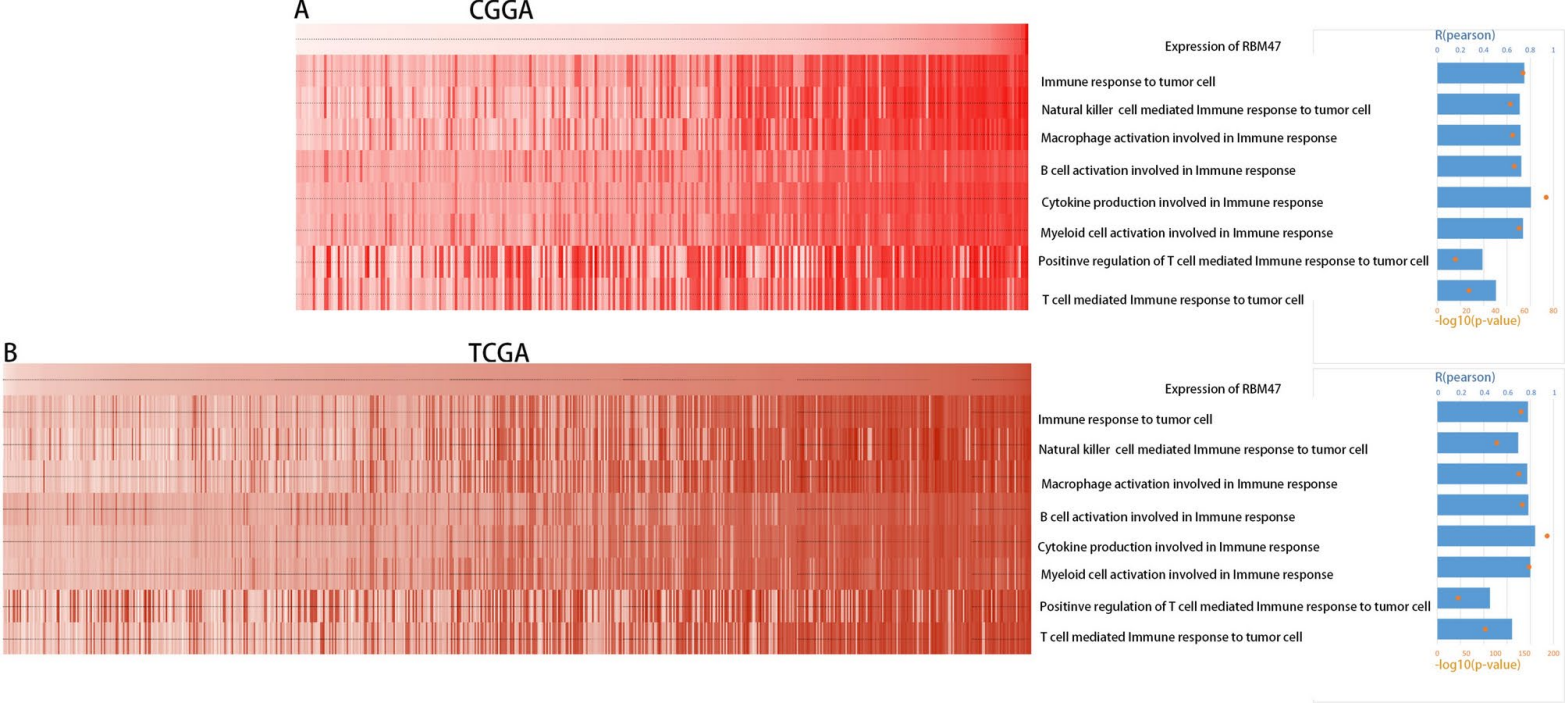
Correlation between RBM47 expression and immune function enrichment score. (**A**) The samples in CGGA were arranged in ascending order of RBM47 expression, and RBM47 expression was associated with immune function enrichment score. The bar chart shows the Pearson-related *R-* and *P*-values. B: TCGA dataset yielded similar results.

Next, we evaluated the relationship between RBM47 expression and various immune processes. We conducted Gene Set Variation Analysis (GSVA), and the results demonstrated that RBM47 expression positively correlated with various immune processes (Fig. 4A,B). To further elucidate the potential immunomodulatory functions of RBM47, we investigated the relationship between its expression levels and those of various known inhibitory immune checkpoints, including programmed cell death protein 1 (PD-1), T-cell immunoglobulin and mucin-domain containing-3 (TIM-3), CD161, cell surface molecule CD200R1, cytotoxic T-lymphocyte-associated protein 4, herpesvirus entry mediator, and T-cell immunoreceptor with Ig and ITIM domains (Table 2). The expression levels of RBM47 showed a strong positive correlation with those of the inhibitory immune checkpoints. The correlation analysis between RBM47 and TIM-3 in two major datasets showed consistent results. In the TCGA dataset, the correlation coefficient between RBM47 and TIM-3 was 0.799 (*p* = 1.5E-73), while in the CGGA dataset, it was 0.844 (*p* = 1.2E-191). These results suggest that RBM47 enhances tumor immune evasion by promoting immunosuppression in the glioma microenvironment.

**Table 2:** Correlation between RBM47 and immunosuppressive checkpoints in CGGA and TCGA datasets.

CGGA			TCGA		
	cor	pvalue		cor	pvalue
RBM47	NA	NA	RBM47	NA	NA
CTLA4	0.204423	0.000207	CTLA4	0.456086	2.34E-37
HVEM	0.609312	2.06E-34	HVEM	0.604365	4.04E-71
TIGIT	0.178596	0.001224	TIGIT	0.235322	2.74E-10
CD200R1	0.411617	1.01E-14	CD200R1	0.530851	2.74E-52
CD161	0.413675	7.24E-15	CD161	0.562126	1.00E-59
PD-1	0.442708	4.96E-17	PD-1	0.590697	3.01E-67
TIM-3	0.799709	1.50E-73	TIM-3	0.844092	1.20E-191

### RBM47 is associated with macrophages in the glioma microenvironment

**Fig. 5:**
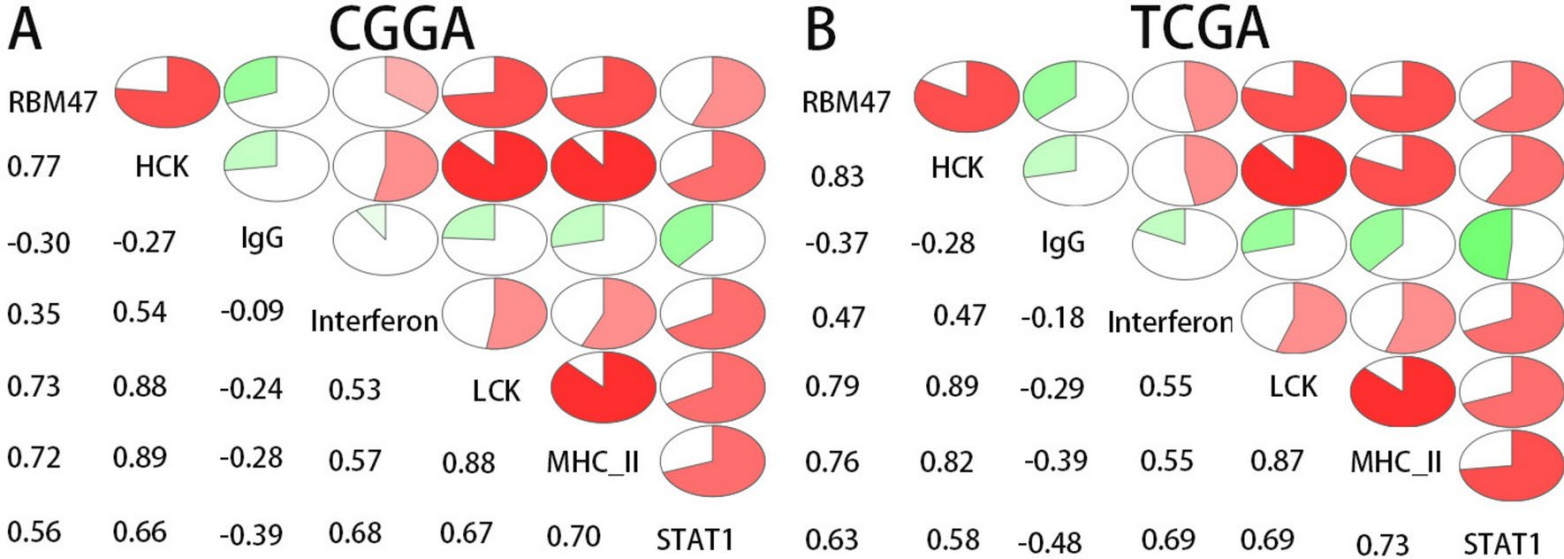
Correlation between RBM47 and inflammation-related metagenes. (**A** and **B**) The CGGA and TCGA datasets showed that RBM47 positively correlates with most inflammatory metagenes, except for IgG.

**Fig. 6:**
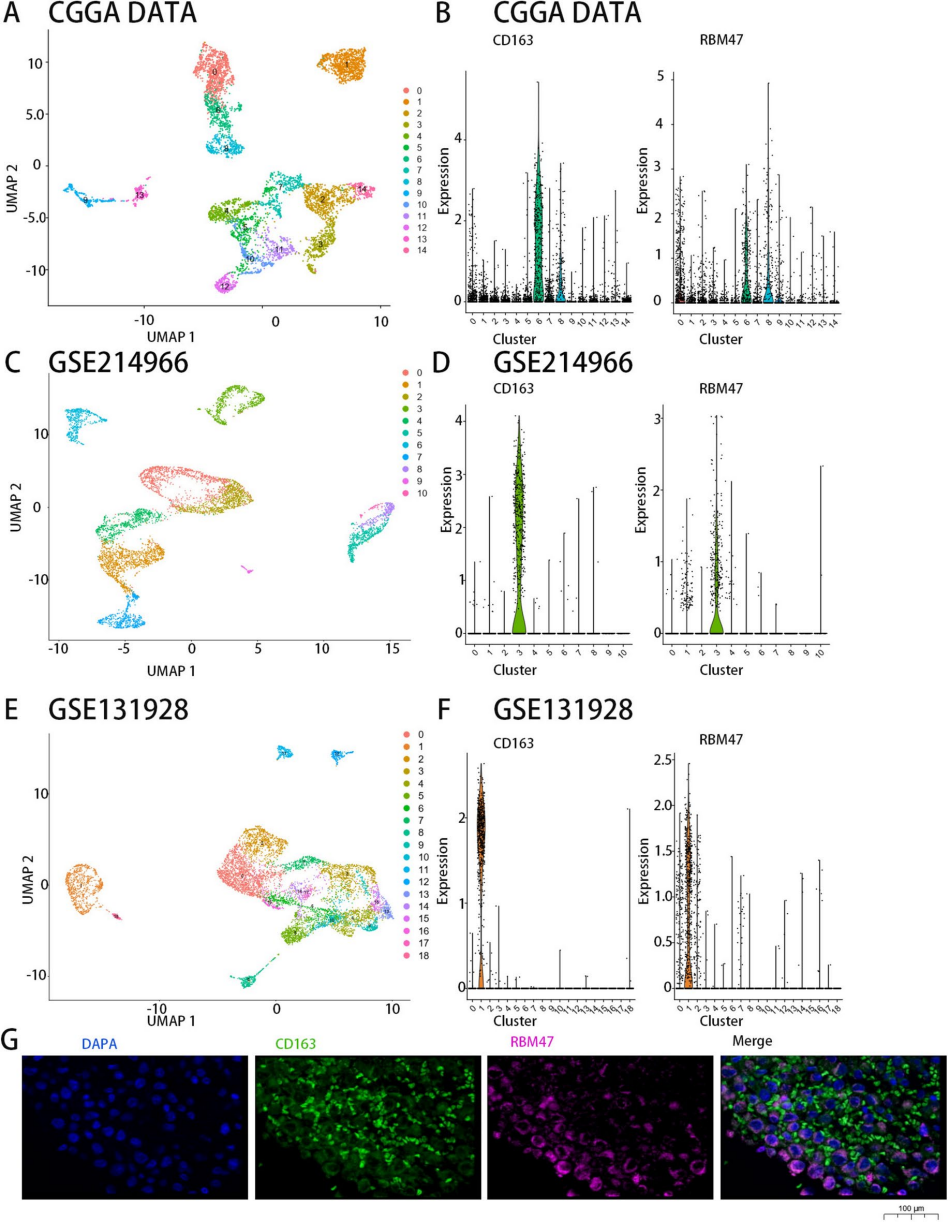
Co-expression of RBM47 and CD163 + macrophages. (**A**, **C**, **E**) UMAP analysis of single-cell sequencing. (**B**) In the CGGA dataset, the M2 macrophage marker CD163 was enriched in clusters 6 and 8, and RBM47 was also enriched in these two clusters. (**D**) In the GSE214966 dataset, the M2 macrophage marker CD163 was enriched in cluster 3, and RBM47 was also enriched in this cluster. (**F**) In the GSE131928 dataset, the M2 macrophage marker CD163 was enriched in cluster 2, and RBM47 was also enriched in this cluster. (**G**) Immunofluorescence showed CD163 and RBM47 co-expression in glioma samples.

To gain further insights into the function of RBM47 in the immune microenvironment of gliomas, we explored the relationship between RBM47 and the inflammatory response. To this end, we analyzed the correlation between six metagenes (List of genes with similar functions in immune cells) and RBM47^9,26,27^. Both the TCGA and CGGA databases yielded remarkably similar results: RBM47 expression was reliably positively correlated with the expression of hematopoietic cell kinase, interferons, lymphocyte-specific protein tyrosine kinase, major histocompatibility complex class II, signal transducer and activator of transcription 1 (STAT1), and STAT2, and negatively related to immunoglobulin G (IgG) expression levels (Fig. 5A,B). These results indicate that RBM47 is involved in inflammatory activation within the tumor. The above results revealed a significant correlation between RBM47, immunity, and inflammation, suggesting that RBM47 likely plays an important role in the immune microenvironment. To gain a more comprehensive understanding of the immune cell types intimately associated with RBM47, we analyzed the single-cell sequencing data from the CGGA dataset. We selected CD163 as an M2 macrophage marker, and the single-cell sequencing results showed that clusters 6 and 8 were M2 macrophages. Unexpectedly, RBM47 was enriched in M2 macrophages (Fig. 6A,B). Validation using the GSE214966 and GSE131928 datasets revealed that, without exception, RBM47 was enriched in M2 macrophages (Fig. 6C–F). Subsequently, we immunofluorescently stained tumor samples and found a co-expression pattern of CD163 and RBM47 within the glioma samples, suggesting a potential interaction between the two (Fig. 6G). Our results suggest that RBM47 reshapes the immune microenvironment of gliomas by modulating M2 macrophages, thereby affecting the prognosis of patients with glioma, making it a potential immunotherapy target.

### RBM47 predicts poorer survival outcomes in glioma

**Fig. 7:**
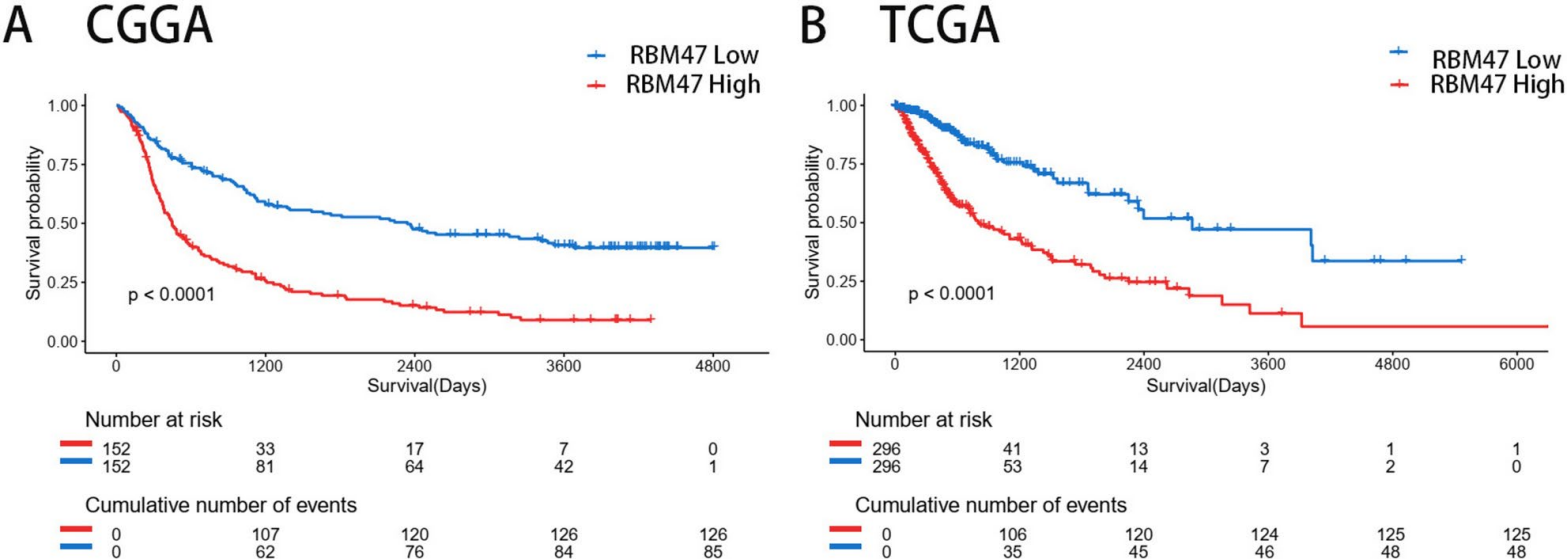
(**A** and **B**) Kaplan–Meier survival curve analysis of CGGA and TCGA datasets showing that high RBM47 expression is associated with poor prognosis.

The aforementioned findings disclosed the crucial function of RBM47 within the immune microenvironment of gliomas. Next, we investigated the prognostic value of RBM47. The Kaplan–Meier method was used to analyze the prognosis of patients based on the CGGA and TCGA databases. The results showed that RBM47 overexpression significantly predicted poorer overall survival (Fig. 7A,B).

Cox regression analyses, encompassing both uni- and multivariate approaches, were performed. The single-factor Cox regression analysis of the CGGA dataset indicated that high WHO grades, older age, and elevated RBM47 expression were associated with poor prognosis. In the multivariate Cox regression analysis, high WHO grades, age, and high RBM47 expression were identified as independent predictive variables (Table 3). Similar results were observed in the analysis of TCGA dataset (Table 4).

**Table 3:** Uni- and multivariate Cox regression analysis of overall survival prognosis in CGGA dataset.

Variable	Univariate analysis		Multivariate analysis	
	HR (95% Cl)	P-value	HR (95% Cl)	P-value
WHO grade				
G2	Reference			
G3	3.576 (2.338–5.471)	< 0.001	3.399 (2.204–5.242)	< 0.001
G4	8.74 (5.881–12.988)	< 0.001	5.916 (3.842–9.110)	< 0.001
Gender	0.934 (0.708–1.232)	0.631		
Age	1.032 (1.020–1.046)	< 0.001	1.014 (1.002–1.026)	0.027
IDH	0.355 (0.268–0.471)	< 0.001	1.213 (0.861–1.708)	0.27
1p19q	0.170 (0.104–0.277)	< 0.001	0.268 (0.158–0.454)	< 0.001
MGMT	0.868 (0.659–1.144)	0.315		
RBM47 expression	1.122 (1.088–1.157)	< 0.001	1.047 (1.010–1.086)	0.013

*P* < 0.05 is considered significantly different.

**Table 4:** Uni- and multivariate Cox regression analysis of overall survival prognosis in TCGA dataset.

Variable	Univariate analysis		Multivariate analysis	
	HR (95% Cl)	P-value	HR (95% Cl)	P-value
WHO grade				
G2	Reference			
G3	3.297 (1.996–5.447)	< 0.001	2.106 (1.249–3.552)	0.005
G4	20.691 (12.401–34.521)	< 0.001	3.391 (1.770–6.496)	< 0.001
Gender	1.007 (0.744–1.364)	0.962		
Age	1.077 (1.064–1.090)	< 0.001	1.061 (1.044–1.078)	< 0.001
IDH	0.090 (0.063–0.128)	< 0.001	0.435 (0.245–0.775)	0.005
1p19q	0.220 (0.130–0.375)	< 0.001	0.742 (0.382–1.442)	0.379
MGMT	0.306 (0.219–0.426)	< 0.001	0.821 (0.558–1.207)	0.316
RBM47 expression	1.721 (1.516–1.953)	< 0.001	1.334 (1.136–1.566)	< 0.001

*P* < 0.05 is considered significantly different.

### A prediction model with improved accuracy in prognostication

**Fig. 8:**
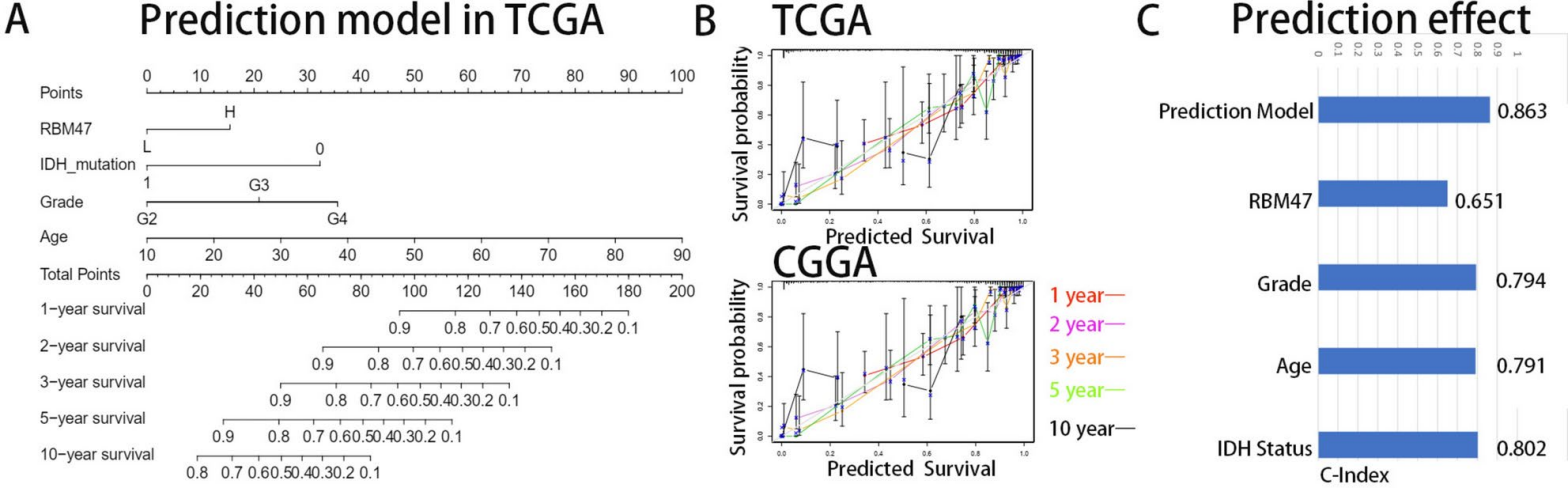
Prediction model. (**A**) Nomogram of RBM47 and other prognostic factors in TCGA database. (**B**) Calibration chart showing the predicted survival probabilities in the training and validation groups. (**C**) C-index evaluation of prediction model’s predictive performance.

Next, we constructed an individualized prediction model. The dataset was split into high- and low-expression groups according to the median RBM47 expression. For the training group, TCGA dataset was used to construct a prediction model incorporating independent predictive factors, such as RBM47 expression and age. Individualized prediction models could estimate the 1-, 2-, 3-, 5-, and 10-year survival rates of patients with low-grade glioma (Fig. 8A). Using the CGGA dataset as the validation group, the calibration curves showed good overlap, indicating good prediction accuracy (Fig. 8B). The C-index of the prediction model was 0.863, indicating that our model was relatively robust (Fig. 8C).

## Discussion

Glioma is one of the most formidable malignant tumors. To date, a few treatment plans that can greatly improve the therapeutic benefits for patients have been identified^28^. Although research has underscored the substantial influence of the tumor microenvironment on tumor progression and patient prognosis, its specific role in glioma progression remains an area that necessitates further investigation^29,30^. Macrophages, fibroblasts, and various non-tumor cells, such as other immune cells, comprise the immune microenvironment^29,31^. Among these, macrophages significantly contribute to glioma proliferation and migration^32^. Meanwhile, previous studies have suggested that CD163 could serve as a potential immunotherapy target for gliomas^33,34^.

In the present study, we used a large number of samples from three datasets: TCGA, CGGA, and GSE4290, GSE214966, and GSE131928 datasets. We conducted immunohistochemistry on glioma paraffin sections and demonstrated that RBM47 is highly expressed in gliomas and is positively correlated with the degree of tumor malignancy. In addition, RBM47 was enriched in wild-type IDH and 1p/19q co-deletion glioma samples, subtypes representing gliomas with poor prognosis and high malignancy. This means that RBM47 may be involved in the malignant progression of gliomas. Further analysis revealed that RBM47 was positively correlated with immunity-related biological processes, which we validated. Moreover, its expression was positively associated with that of several known inhibitory immune checkpoints. Specifically, the correlation coefficient with TIM-3 reached 0.799 (*p* = 1.5E-73) in the first dataset and 0.844 (p = 1.2E-191) in the second dataset. TIM-3 is also expressed by macrophages^35^, and TIM-3 inhibitors have been used clinically to treat several tumors^36,37^.

Next, we analyzed CGGA single-cell sequencing data and found that RBM47 was enriched in CD163 + macrophages. We also validated the co-expression of CD163 and RBM47 in glioma samples using immunofluorescence. Previous studies have shown that RBM47 knockdown in U87 and U251 glioma cell lines inhibits the polarization of M2 cells in vivo and in vitro^21^. In addition, the same authors demonstrated that RBM47 is a pro-oncogene, corroborating the results of the present study. However, our single-cell sequencing data analysis revealed that RBM47 was predominantly expressed in M2 macrophages and was partially expressed in glioma cells. Accordingly, RBM47 likely regulates the immune microenvironment in gliomas by modulating M2 macrophages.

Immune checkpoint therapy has revolutionized the treatment landscape of several solid tumors. A future development trend might involve combining different immune checkpoints as part of a comprehensive treatment strategy. Research has found that combination therapy with anti-programmed cell death protein 1, anti-TIM-3, and focal radiotherapy achieved superior results in mouse gliomas^38^. The phenotype of macrophages mediated by CD73 in glioblastoma multiforme has immunosuppressive effects, affecting immune checkpoint therapy^39^. Combination therapy targeting CD73 with dual blockade of PD-1 and CTLA-4 has achieved better therapeutic effects in mouse models^39^. Considering the immune role of RBM47 in gliomas, it may be a candidate for combination therapy with immune checkpoint inhibitors such as TIM-3 and PD-1. Currently, no research reports exist on effective therapeutic targeted drugs for RBM47. This gap poses a significant challenge that requires further research.

Furthermore, elevated RBM47 expression levels have been linked to an unfavorable prognosis. Multivariate regression analysis verified RBM47 as an independent prognostic factor. Next, we constructed a nomogram based on the expression and clinical features of RBM47 in TCGA dataset to more accurately predict overall survival. The model was effectively validated using the CGGA dataset with a C-index of 0.863. Therefore, RBM47 may serve as a potential prognostic factor in patients with glioma.

This study is not without limitations. Our research only included limited in vitro experimental validation, necessitating more comprehensive in vivo validation. By co-culturing glioma cell lines with induced M2 macrophages and subsequently subjecting the macrophages to RBM47 knockdown, we can observe the proliferation, invasion, and migration abilities of tumor cells, which will help to understand the function of RBM47.

In conclusion, this multi-dataset study, coupled with immunohistochemical analyses, revealed that RBM47 is highly expressed in gliomas and is correlated with increasing malignancy. RBM47 appears to promote an immunosuppressive tumor microenvironment through M2 macrophage enrichment and its association with inhibitory immune checkpoints. RBM47 overexpression is an independent predictor of poor prognosis in patients with glioma. Furthermore, an individualized RBM47-based prediction model demonstrated robust prognostic accuracy. Combining traditional treatments with immune-targeted therapy for gliomas is a promising therapeutic option to improve patient prognosis. Therefore, we propose RBM47 as a potential immunotherapeutic target and prognostic biomarker for gliomas, though further mechanistic validation is needed.

## Data Availability

The datasets utilized in this study are publicly available in online repositories. The CGGA dataset for 325 samples was sourced from http://www.cgga.org.cn/mRNAseq_325. The TCGA dataset for 702 samples was sourced from https://cancergenome.nih.gov. The GSE4290, GSE214966, and GSE131928 datasets were sourced from https://www.ncbi.nlm.gov/geo, and the login numbers are GSE4290, GSE214966 and GSE131928.
